# Evaluation of a health education program for improving uptake of HIV self-testing by men in Rwanda: a pilot pragmatic randomized control trial

**DOI:** 10.1186/s40814-021-00940-x

**Published:** 2021-11-12

**Authors:** Tafadzwa Dzinamarira, Claude Mambo Muvunyi, Tivani Phosa Mashamba-Thompson

**Affiliations:** 1 Department of Public Health Medicine, School of Nursing and Public Health, University of KwaZulu-Natal, Durban, 4001 South Africa; 2 College of Medicine and Health Sciences, University of Rwanda, Kigali, Rwanda; 3 CIHR Canadian HIV Trials Network, Vancouver, BC Canada; 4 Faculty of Health Sciences, University of Pretoria, Pretoria, Pretoria South Africa

**Keywords:** Health education, Men, HIV self-testing

## Abstract

**Background:**

Health education interventions tailored to suit men have the potential to improve health outcomes for this underserved population. HIV self-testing (HIVST) is a promising approach to overcoming challenges associated with low HIV testing rates among men. The primary objective of this study is to assess the feasibility of conducting a definitive trial to determine the effectiveness of a locally adapted and optimized health education program (HEP) on the uptake of HIVST among men in Kigali, Rwanda.

**Methods:**

This study employs a pilot pragmatic randomized controlled trial to evaluate an HIVST HEP for men. Participants were randomized to the intervention (HEP) arm or to the control arm. In the intervention group, the adapted HEP was administered in addition to routine health education. In the non-intervention group, only routine health education was offered. Participant data was collected first upon recruitment and then after 3 months’ follow-up using interviewer-administered questionnaires.

**Results:**

There was a 100% response rate at enrollment and no loss to follow-up at exit. There was significant association between the study arm and knowledge of HIVST. Participants in the control arm had a mean knowledge score of 67% compared to 92% among participants in the intervention arm. There was an association between the study arm and HIVST uptake: 67% of the study participants in the intervention arm self-reported HIVST uptake compared to 23% of the participants in the control arm.

**Discussion:**

This pilot study demonstrates the feasibility of a larger trial to assess the effectiveness of an HEP intervention on uptake of HIVST among men. We found preliminary evidence of increased uptake of HIVST in the intervention group.

**Trial registration:**

Pan African Clinical Trial Registry PACTR201908758321490. Registered on 8 August 2019.

**Supplementary Information:**

The online version contains supplementary material available at 10.1186/s40814-021-00940-x.

## Key messages regarding feasibility

- The pilot study proved a larger trial to implement and assess effectiveness of the health education program intervention is feasible. However, the Hawthorne effect cannot be excluded for this pilot study as use self-report data to guide on feasibility of this trial.
- The intervention can be implemented and assessed in a larger randomized controlled trial. There was 100% enrollment and no loss to follow-up for the pilot study. Participants in the intervention group had a more favorable outcome with regard to uptake of HIV self-testing.
- The design of the main study will require a more objective tool or confirmation for the primary outcome; uptake of HIV self-testing.

## Background

The Joint United Nations Programme on HIV and AIDS (UNAIDS) reports that in 2018, 21% of people living with HIV/AIDS were unaware of their status [1]. Despite the major success reported in the HIV care continuum cascade, knowledge of one’s status remains an important first step to accessing treatment [1, 2]. Rwandan health system generally appears to be stemming the tide of the HIV/AIDS epidemic with a reported decline in HIV prevalence to below 3% [3], from 13% in the 1990s [4]. Major success has been reported in the HIV response in Rwanda; however, available evidence shows a gap between the current situation and the UNAIDS 90-90-90 target for 2020 [3]. Addressing male aversion to HIV testing services (HTS) has remained a priority in Rwanda’s HIV program for more than two decades now [4, 5], since a gap in testing rates between men and women has been consistently observed in national surveys conducted in 2005 [6], 2010 [7], 2015 [8], and 2019 [3].

Men have been reported to fear confidentiality breaches due to not trusting healthcare workers [9–11], so HIV self-test kits enable individuals to test themselves for HIV in their home, without the presence of healthcare providers [12, 13]. HIV self-testing (HIVST), therefore, may potentially benefit men who routinely experience significant breaches to health care anonymity and confidentiality [14–16]. While a number of benefits are associated with HIVST, long-standing barriers to accessing HTS are likely to remain pertinent [17]. A qualitative study conducted on health officials, community health workers, and persons living with HIV in South Africa revealed that people who do not feel “ready” to know their status, who worry about HIV-related stigmas, or who fear dying of AIDS will possibly still be reluctant to be tested, even when self-test kits become available [18]. The main reported concerns about HIVST uptake also include a general lack of counseling, accuracy hindrances and a potential for the coercive use of self-testing devices [16, 18, 19]. Provision of health education has been recommended to ensure the proper uptake of HIVST [17, 20, 21].

Health education is one of the main strategies that can be used to ensure the effective implementation of health promotion and disease prevention programs [22]. Major success has been reported when health education strategies are tailored to their target population [22]. Delivery models for health education vary between classes, workshops, webinars, lectures, and courses [23], which present information through audiovisual and computer-based supports like projectors, slides, books, videos, posters, CDs, websites, pictures and software programs [24]. A key characteristic of a successful health education strategy is a carefully planned learning activity that is well informed and aimed at increasing the skills and knowledge of the participants [25]. There is global evidence that health education programs improve health outcomes. Health education interventions about voluntary circumcision and HIV among youth in Kenya [26], male involvement in family planning in Malawi [27] and India [28], and male involvement in maternity care in South Africa [29], Nepal [30], and Pakistan [31] all reported improved health outcomes.

This study is part of a larger study [32] aimed at employing an HEP for improving men’s uptake of HIVST. In the earlier phases of this study, we conducted interviews with key stakeholders to assess their perspectives on the implementation and upscaling of HIVST in Rwanda [33]. A cross-sectional study was employed to assess awareness and acceptability of HIVST among men in Kigali, Rwanda [34]. Guided by the findings from the interviews with stakeholders and from the cross-sectional study, we employed the nominal group technique in a co-creation workshop involving key stakeholders in the HIV response in Rwanda to design the health education program. A detailed account of the methodology and the results of the workshop are fully described in a manuscript submitted elsewhere [35]. A two-step process was followed to identify priority barriers to men’s uptake of current HTS and to co-create, with key stakeholder, an HEP for HIVST that would address these barriers. The outcome was a male-tailored health education program aimed at improving HIVST uptake. The current study assesses the feasibility of conducting a larger trial. This trial would evaluate the locally adapted and optimized health education program designed to improve the uptake of HIVST among men in Kigali, Rwanda.

## Methods

### Design

This study is part of a multi-phase PhD project; protocol for the main study is published elsewhere [32]. The research group conducted a two-arm pilot pragmatic randomized control trial. This trial was registered in the Pan African Clinical Trial Registry, PACTR201908758321490), https://pactr.samrc.ac.za/TrialDisplay.aspx?TrialID=8310.

In this trial, participants were randomized to the health education program arm (HEP) or to the control arm. In the intervention group, the adapted HEP was administered in addition to routine health education. Data collection occurred through interviewer-administered questionnaires that were transferred onto mobile tablet devices using the pre-programmed study software, Open Data Kit (ODK).

### Study setting

The University Teaching Hospital of Kigali (CHUK) was purposively selected to pilot the implementation the HEP intervention. CHUK is the largest hospital located in Nyarugenge District, Kigali City. It is also the largest referral hospital in the country. Trained health professionals introduced the intervention in the outpatient department, as outpatients are not expected have critical conditions that would affect their ability to take part in the HEP or the interviewer-administered questionnaire.

#### Inclusion criteria

This study included participants who met the eligibility criteria below:

- Adult male, 18 years and older
- Unknown HIV status
- Is visiting the study-selected health facility during the enrolment period
- Is willing to be followed up with three months post-enrolment

#### Exclusion criteria

This study excluded participants who met the eligibility criteria below:

- Female gender
- Men younger than 18 years of age
- Known HIV status
- Is unwilling to be followed up with three months post-enrolment

### Control

The control group received routine health education on HIV/AIDS and sexual health. This was delivered in Kinyarwanda, the local language, by trained health professionals. The sessions lasted approximately 45 min.

### Intervention

The intervention, an HEP, was an interactive, structured curriculum containing eight modules with information on health locus of control, HIV etiology and transmission, diagnosis, status disclosure benefits, and care and treatment services. The modules also included an overview of the background of HIVST and the testing procedure. The contents of the HEP were validated by the HIV Division in the Rwanda Biomedical Center, Ministry of Health. The HEP was delivered through face-to-face communication and using information education communication materials. The HEP was taught in a maximum of 1 h. Two health professionals at the study site were trained to deliver the HEP in Kinyarwanda. Follow-up interviews were scheduled for 3 months post-enrolment.

### Outcomes

#### Primary outcome(s)

The main outcome of this study is feasibility of a larger RCT trial measured by recruitment into the study and uptake of HIVST. The feasibility criteria were measured by number of participants retained at follow-up for an exit interview. For the purposes of this trial, uptake is defined as self-reported use of the HIVST kit at the follow-up interview.

#### Secondary outcome(s)

This study also assessed HIV diagnosis among participants, linkage to care among men who tested HIV positive, 3-month repeat tests for men who tested HIV negative, and HIV status disclosure to sexual partner(s) after HIV self-testing.

### Hypothesis of the larger pragmatic trial

The locally adapted and optimized health education program will improve the uptake of HIVST in the intervention arm as compared to routine health education in the control arm among men in Kigali, Rwanda.

### Sample size

For this study, we recruited 60 men who were randomized in a 1:1 ratio between the intervention and control groups. Given that this was a pilot study, we followed the recommendation of Browne et al. [36] to determine the sample size based on the estimated parameter of 30 participants.

### Recruitment of participants

The study’s population was men attending the study site who self-reported unknown HIV status at the time of enrolment, were above the age of 18 years, and were residents in Kigali Province during the period of March–July 2020. The target population was men who were visiting the study’s health facility as clients, were accompanying or visiting relatives, or were visiting for other reasons.

### Randomization procedure and blinding

We employed simple randomization using a randomization table created by a computer software program (RALLOC, STATA) and an independent statistician. The researcher kept sequentially numbered allocation codes in sealed envelopes. Each eligible participant collected an envelope containing their allocation after baseline data has been completed. This method prevented participants and recruiters from knowing the study group to which the next participant would be assigned [37]. We chose this approach, as it helped to ensure that a participant’s decision to provide informed consent, or a recruiter’s decision to enroll a participant, was not influenced by knowledge of the group to which they would be allocated if they joined the trial [37, 38]. We conducted an open-label trial, meaning participants were aware of their study arm.

### Data collection methods

At enrolment, participants’ baseline data was captured using an interviewer-administered questionnaire (Supplementary File 1). We pilot-tested the data collection tool on ten non-survey participants to assess clarity of questions, their reliability, and their validity. Participants in the control group received routine health education on HIV/AIDS and sexual health. In addition to routine health education, participants in the intervention group received the HEP (Supplementary File 2). At the follow-up interview, we employed an interviewer-administered questionnaire (Supplementary File 3) for data collection. Quality assurance during the trial was secured through the effective supervision of the trained data collectors. The first (TD) and second (CMM) authors were responsible for providing supervision to the two research assistants during enrolment, baseline data collection, intervention administration, and follow-up interview data collection. All data was captured on a pre-programmed study software, ODK, with transmission after each participant. Knowledge, attitudes, and practice were assessed using a set of questions: knowledge (16 questions), attitude (12 questions), and practice (11 questions)

### Data management

In this study, all data was entered electronically. The data entry screens resembled the paper forms approved by the institutional review boards. We ensured data integrity through a variety of mechanisms. Validation tools were built into the tablet data collection system to minimize data entry errors and to encourage proper form completion. Pre-defined values (code sets) for categorical data, range restrictions for numeric data, and logic checks were used to restrict the type of data and minimize data entry errors. Entry of data in the key fields was required to ensure completeness, and skip patterns were used to prevent irrelevant data entry. The software was equipped with error messages and caution notices that would be triggered if data collection staff were to enter faulty data. All the study’s paper forms were kept in locked cabinets, and access to the study data was restricted. A password system was utilized to control access to the tablets used for data collection.

### Data analysis

Feasibility outcomes, determined by recruitment and retention in the trial were calculated by using raw count numbers and percentages. A quality score was calculated for each of the knowledge, attitude, and practice domains. The three domain scores were independent and hence not aggregated into a single quality score. The domain scores were calculated by totaling the scores of the individual items in a domain and by scaling the total as a percentage of the maximum possible score for that domain. The scaled domain score = (obtained score – minimum possible score)/(maximum possible score – minimum possible score). We used the chi-square test to determine association between categorical dependent and categorical independent variables. We used the Student *t* test to determine association between quantitative dependent and categorical independent variables. All statistical tests were performed in the STATA software package. A *p* value < 0.05 was considered significant.

## Results

### Participant demographic and sexual health history characteristics

**Fig. 1:**
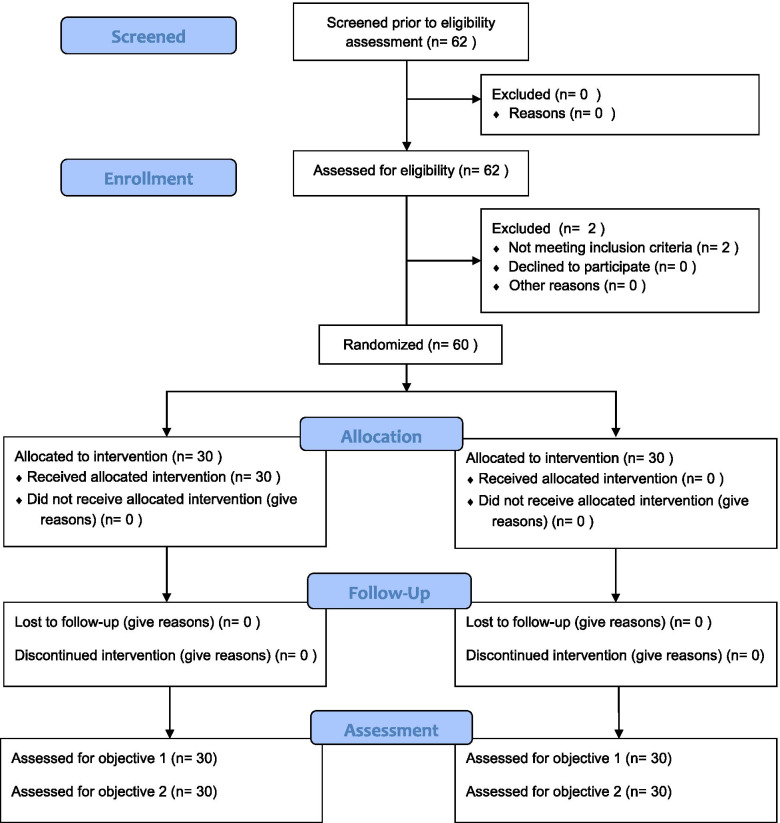
Flow diagram

A total of 60 participants, randomized into either the control arm (50%) or the intervention arm (50%), were enrolled for this pilot study. There was a 100% response rate at enrolment and no loss to follow-up at exit (Fig. 1). The mean age of the participants was 32 years. Nineteen percent was enrolled from Gasabo District, 32% from Nyarugenge District, and 28% from Kicukiro District. Twenty-eight percent were single, 50% were married, and the rest were either cohabiting, widowed, or divorced. More details on demographic characteristics are available in Table 1.

**Table 1:** Participant demographic characteristics

Variable	Response (***n*** = 60)
Age in years, mean (SD)	32.0 (9.8)
Education level, *n* (%)	
Primary	6 (10)
Secondary	28 (47)
Tertiary	25 (42)
Did not attend school	1 (2)
Income, *n* (%)	
Self-employed	22 (37)
Professional	23 (38)
Unemployed	15 (25)
District, *n* (%)	
Gasabo	24 (19)
Nyarugenge	19 (32)
Kicukiro	17 (28)
Marital status, *n* (%)	
Single	17 (28)
Cohabitation	8 (13)
Widowed	4 (7)
Married	30 (50)
Divorced	1 (2)
Religion, *n* (%)	
Christian	49 (82)
Muslim	10 (17)
Other specify	1 (2)

All the participants reported to have had sexual intercourse. The majority (93%) were heterosexual, while 7% were homosexual. The median number of each participant’s sexual partners over last 12 months was one, ranging from one to six partners. More details are presented on participant sexual characteristics in Table 2.

**Table 2:** Sexual health history and health seeking behavior

Variable	Response
Ever had sexual intercourse, *n* (%)	
Yes	60 (100)
Sexual preference, *n* (%)	
Heterosexual	56 (93)
Homosexual	4 (7)
Number of different sexual partners last 12 months, median (range) an (SD)	1 (1 – 6)
Ever paid for sex 1 month ago, *n* (%)	
Yes	19 (32)
Circumcised, *n* (%)	
Yes	30 (50)
Know condom use, *n* (%)	
Yes	60 (100)
Frequency condom use last 12 months, *n* (%)	
Never	12 (20)
Rarely	48 (80)
Risk of HIV, *n* (%)	
Fairly high	6 (10)
Low	14 (23)
Very low	40 (67)
Last visit to health facility, *n* (%)	
Never	18 (30)
1–3 months ago	22 (37)
4–6 months ago	1 (2)
6–12 months ago	2 (3)
More than a year ago	17 (28)
Last 3 months sick, *n* (%)	
Consultation with qualified medical practitioners	18 (30)
Consultation with community health worker	22 (37)
Consultation with traditional health care practitioners	1 (2)
Consultation with over the counter drug sellers	2 (3)
Consultation with self and other family members	17 (28)

### Knowledge, attitude, and perception toward HIVST

There was a significant difference in the change of knowledge level between the control and intervention, the control had an average increase in the knowledge score of 7.1 compared to the intervention group which had an average increase of 30.6. There was a significant difference in the change of attitude level between the control and intervention; the control had an average decrease in the attitude score of 2.1 compared to the intervention group which had an average increase of 14.8. There was a significant difference in the change of perception level between the control and intervention; the control had an average decrease in the perception score of 4.9 compared to the intervention group which had an average increase of 17.7. Details on mean knowledge score changes are presented in Table 3.

**Table 3:** Analysis of change in knowledge, attitude, and perceptions toward HIVST from enrolment to follow-up across study groups

Variable	Control	Intervention	Difference
Knowledge score, mean difference% (95% CI)	7.1 (2.5–11.7)	30.6 (23.5–37.8)	23.5 (15.2–31.9)
Attitude score, mean difference% (95% CI)	− 2.1 (− 7.6–3.5)	14.8 (5.5–25.3)	16.9 (6.3–27.6)
Perception score, mean difference% (95% CI)	− 4.9 (− 10.5–0.6)	17.7 (0.9–11.9)	22.6 (13.4–31.9)

### Self-reported HIV testing at follow-up visit

There was an association between having tested for HIV and the study arm (*p* < 0.05): in the intervention arm, 67% of the participants had received an HIV test; in the control arm, 27%.

There was no association between the study arm and the following: the number of times a participant reported to have been tested for HIV at the exit, the place the first HIV test was performed, the place last HIV test was performed, and the first HIV test result (*p* > 0.05). More details are presented in Table 4.

**Table 4:** Self-reported HIV testing at follow-up

Variable	Control	Intervention	Difference % (95% CI)
Ever tested for HIV, *n* (%)			
No	22 (73)	10 (33)	34 (22–46)
Yes	8 (27)	20 67)	Ref
Number of times have you been tested for HIV, *n* (%)			
1	5 (28)	13 (72)	6 (− 3–15)
2	2 (22)	7 (78)	Ref
Where was the first HIV test done since our last discussion?			
Home	7 (26)	20 (74)	74 (58–90)
Health facility	1 (100)	–	Ref
Last HIV test done since our last discussion, *n* (%)			
Home	1 (25)	3 (75)	8 (− 9–25)
Health facility	2 (33)	4 (67)	30 (13–47)
First HIV test result, *n* (%)			
Positive	–	1 (100)	Ref
Negative	8 (30)	19 (70)	34 (22–46)

## Discussion

This study has determined the feasibility of conducting a larger trial to evaluate the effectiveness of a male-tailored health education program on the uptake of HIVST among men in Kigali, Rwanda. Men’s engagement in health services, particularly HIV testing services, is a priority in Rwanda’s public health agenda [8]. To address this priority, key stakeholders in HIV response in Rwanda have called for swift implementation and evaluation of HIVST intervention [33]. This is consistent with the World Health Organization’s recommendation to evaluate the interventions used to upscale HIVST uptake among different groups in terms of gender, socio-economic status, and education level [39].

In this pilot study, there was 100% recruitment at enrolment and no loss to follow-up at exit. There was a marked difference in mean knowledge among the two different arms of the study, with a higher mean knowledge (92%) in the arm that received the intervention compared to a lower mean knowledge (62%) in the control arm. A systematic review by Pant Pai et al*.* reveals that knowledge on HIVST drives the motivation that improves the uptake of HIVST [40]. This has also been evidenced by a study in South Africa, after the introduction of an HIVST network, that helped educate men about HIVST [41]. Our earlier work on the awareness and acceptability of HIVST in Rwanda found low awareness and high acceptability of HIVST [34]. The results of the current pilot study reveal a need for a health education intervention to improve men’s knowledge on HIVST intervention. Studies conducted in Botswana [42] and Tanzania [21] have revealed similar gaps in the knowledge of HIVST among men. An HIVST strategy that addresses the concerns of the male population is likely to be feasible for and relevant to the Rwandese male population. This study found an association between attitude toward HIVST and uptake; in the control arm, participants showed a mean attitude score of 65%; in the intervention arm, 84%. Similar findings were reported among men who have sex with men in the Philippines after an introduction of an HIVST strategy focused and targeted on them and tailored for men [43]. These findings are promising concerning the establishment of a full-scale, larger trial for the intervention. At present, there is limited evidence on the effectiveness of health education programs on HIVST uptake among men. However, a number of studies have drawn conclusions on the potential of education programs to improve HIVST uptake among priority populations, including men. A study on oral HIVST uptake among men in Uganda revealed that the participants could not comprehend how HIV testing could be done without using a blood sample. The study revealed a need to intensify health education about oral HIVST to improve uptake [44]. A study conducted in Nepal recommended the use of a one-to-one education approach in providing HIVST information [45].

A major strength of this study is its implementation of the intervention in a pragmatic approach. Consistent with the nature of pragmatic trials, this study’s findings are applicable to routine practice. However, this study has its limitations. Though recruited through methods designed to generate a representative sample, as this is a pilot study, the sample is unlikely to be representative of all men in Kigali. Additionally, the sample was largely young with a mean age of 32 years, which indicates an underrepresentation of older men. Since this was a pilot study, the sample size determination was small. Some inferential statistics were performed in the study to only get an insight of variables that are likely to have an association; however, some if not all the statistical tests were subject to huge bias because of the small sample size used in performing these tests. It is therefore apparent that some statistical significance association (*p* < 0.05) observed in the study only means that there is a possibility of an association that can only be concluded or confirmed by the main study. Therefore, clinical outcomes from this feasibility trial are suggestive rather than definitive. While the results from this study will add to the ever-growing data that HIVST would upscale HIV testing, the limitations of the study warrant larger in-depth, randomized controlled trials for understanding the impact of the health education program on HIVST uptake.

## Conclusion

This pilot study demonstrates the feasibility of a larger trial to assess the effectiveness of a health education program intervention on the uptake of HIVST among men. We found preliminary evidence of an increased uptake of HIVST in the intervention group.

## Supplementary Information

**Additional file 1.**

**Additional file 2.**

**Additional file 3.**

## Data Availability

The raw data analyzed in this study is available upon reasonable written request submitted to the corresponding author.

## Abbreviations

HIVST: HIV self-testing
HEP: Health Education Program
ODK: Open Data Kit
