# Supplementary material for: Evaluation of a health education program for improving uptake of HIV self-testing by men in Rwanda: a pilot pragmatic randomized control trial
Source: Pilot Feasibility Stud. 2021 Nov 12;7:202. doi: 10.1186/s40814-021-00940-x (PMC8588608; doi:10.1186/s40814-021-00940-x)
Supplement: Supplementary file 1 — Additional file 1. [file 40814_2021_940_MOESM1_ESM.docx]

**APPENDIX C: ENROLLMENT QUESTIONNAIRE**

**Respondent study ID: ____________________**

Thank you for agreeing to participate in this study. The first sets of questions are about your demographic information, followed by questions related to sexual and health seeking behaviour, HIV- self testing knowledge, attitude and perception. Mark with an X in the appropriate box.

**PART I: DEMOGRAPHIC INFORMATION**

1. Age: ___
2. What is your level of education?

| Did not attend school | Primary | Secondary | Tertiary |
| --- | --- | --- | --- |
| **□** | **□** | **□** | **□** |

1. Source of financial income:

| Unemployed | Self-employed | Professional |
| --- | --- | --- |
| **□** | **□** | **□** |

1. District in which you reside in Kigali:

| Gasabo | Nyarugenge | Kicukiro |
| --- | --- | --- |
| **□** | **□** | **□** |

1. Marital status:

| Single | Cohabitation | Widowed | Married | Divorced/Separated |
| --- | --- | --- | --- | --- |
| **□** | **□** | **□** | **□** | **□** |

1. Religion:

| Christian | Muslim | Other (Specify) |
| --- | --- | --- |
| **□** | **□** | **______________________** |

**PART II: SEXUAL AND HEALTH SEEKING BEHAVIOUR**

1. Have you ever had sexual intercourse?

| Yes | No |
| --- | --- |
| **□** | **□** |

1. What is your sexual preference?

| Heterosexual | Homosexual | Bisexual |
| --- | --- | --- |
| **□** | **□** | **□** |

1. In the last 12 months, with how many different sexual partners did you have? ____
2. Have you ever paid or received payment for sex (payment includes monetary or non-monetary gifts) in the last one month? (Tick one)

| Yes | No |
| --- | --- |
| **□** | **□** |

1. Are you circumcised?

| Yes | No |
| --- | --- |
| **□** | **□** |

1. Do you know how to use a condom?

| Yes | No |
| --- | --- |
| **□** | **□** |

1. How often did you use a condom in the last twelve months?

| Never | Rarely | Sometimes | Always |
| --- | --- | --- | --- |
| **□** | **□** | **□** | **□** |

1. To what extent do you think you are at risk of acquiring HIV?

| Very High | High | Fairly High | Low | Very Low |
| --- | --- | --- | --- | --- |
| **□** | **□** | **□** | **□** | **□** |

1. When was the last time you visited a health facility or professional medical practitioner for an ordinary check-up?

| Never | 1 - 3 months ago | 4 - 6 months ago | 6 – 12 months ago | More than a year ago |
| --- | --- | --- | --- | --- |
| **□** | **□** | **□** | **□** | **□** |

1. The last three times you were sick, which of the following did you do first?

| 1. Consultation with qualified medical practitioners | **□** |
| --- | --- |
| 1. Consultation with community health worker | **□** |
| 1. Consultation with traditional health care practitioners | **□** |
| 1. Consultation with over the counter drug sellers | **□** |
| 1. Consultation with self and other family members | **□** |
| 1. Nothing | **□** |

**PART III: KNOWLEDGE OF HIV SELF TESTING**

| Number | Question | Strongly Disagree | Disagree | Neutral | Agree | Strongly Agree |
| --- | --- | --- | --- | --- | --- | --- |
| 1 | I have heard about HIVST |  |  |  |  |  |
| 2 | I have seen an HIVST |  |  |  |  |  |
| 3 | I have used an HIVST |  |  |  |  |  |
| 4 | I have read about HIVST |  |  |  |  |  |
| 5 | It is legal to use HIVST kits in Rwanda |  |  |  |  |  |
| 6 | HIVST are available from private pharmacies |  |  |  |  |  |
| 7 | HIVST kits are available in government clinics/hospitals |  |  |  |  |  |
| 8 | HIVST kits are available on the internet |  |  |  |  |  |
| 9 | HIVST is done using blood |  |  |  |  |  |
| 10 | HIVST is done using fluid from the mouth |  |  |  |  |  |
| 11 | A person can perform the HIVST on herself/himself |  |  |  |  |  |
| 12 | It takes 20–40 minutes to get results from the HIVST |  |  |  |  |  |
| 13 | The test can be negative if the HIV infection is less than three months |  |  |  |  |  |
| 14 | A person needs to re-test after three months if the test is negative |  |  |  |  |  |
| 15 | There is a telephone hotline to call should the test be positive |  |  |  |  |  |
| 16 | A person needs to be counselled by the HIV counsellor before taking the HIV self-test |  |  |  |  |  |

**PART IV: ATTITUDE TOWARD HIV SELF TESTING**

| Number | Question | Strongly Disagree | Disagree | Neutral | Agree | Strongly Agree |
| --- | --- | --- | --- | --- | --- | --- |
| 1 | In your opinion; is HIVST is a good idea? |  |  |  |  |  |
| 2 | Do you think you can be able to do an HIVST at home? |  |  |  |  |  |
| 3 | I think I would find the HIV self-testing procedure difficult to perform |  |  |  |  |  |
| 4 | I would prefer to self-test alone |  |  |  |  |  |
| 5 | I would prefer to self-test at the health facility |  |  |  |  |  |
| 6 | I would prefer to self-test with partner |  |  |  |  |  |
| 7 | I would prefer to self-test and read the results myself |  |  |  |  |  |
| 8 | I would like to get telephone counselling before the HIVST |  |  |  |  |  |
| 9 | I would seek help from the clinic should the test be positive |  |  |  |  |  |
| 10 | I would like to get face to face counselling after the test |  |  |  |  |  |
| 11 | It is important to follow up an HIV positive result at the clinic |  |  |  |  |  |
| 12 | It is important to get counselling after the test |  |  |  |  |  |

**PART V: PERCEPTION TOWARD HIV SELF TESTING**

| Number | Question | Strongly Disagree | Disagree | Neutral | Agree | Strongly Agree |
| --- | --- | --- | --- | --- | --- | --- |
| 1 | Privacy is ensured with HIVST |  |  |  |  |  |
| 2 | Less time is spent in clinics and hospitals |  |  |  |  |  |
| 3 | More people can know their status |  |  |  |  |  |
| 4 | People who are scared to go to the clinics can test at home |  |  |  |  |  |
| 5 | There could be less transmission of HIV to other people |  |  |  |  |  |
| 6 | People could be tested more frequently |  |  |  |  |  |
| 7 | People could read/interpret results incorrectly |  |  |  |  |  |
| 8 | People may not be able to read instructions properly |  |  |  |  |  |
| 9 | People could intentionally infect others if not properly counselled before the test |  |  |  |  |  |
| 10 | HIVST could result in people being tested against their will |  |  |  |  |  |
| 11 | Family members could be tested against their will, which could result in abuse |  |  |  |  |  |
